# Open-label, single-center, phase I trial to investigate the mass balance and absolute bioavailability of the highly selective oral MET inhibitor tepotinib in healthy volunteers

**DOI:** 10.1007/s10637-020-00926-1

**Published:** 2020-03-27

**Authors:** Andreas Johne, Holger Scheible, Andreas Becker, Jan Jaap van Lier, Peter Wolna, Michael Meyring

**Affiliations:** 1 Global Clinical Development, Merck KGaA, Frankfurter Strasse 250, 64293 Darmstadt, Germany; 2 Institute of Drug Metabolism and Pharmacokinetics, Merck KGaA, Grafing, Germany; 3 Pharmaceutical Research Association (PRA), Groningen, The Netherlands

**Keywords:** Bioavailability, Mass balance, Metabolites, Pharmacokinetics, Tepotinib, Tyrosine kinase inhibitor

## Abstract

Tepotinib (MSC2156119J) is an oral, potent, highly selective MET inhibitor. This open-label, phase I study in healthy volunteers (EudraCT 2013-003226-86) investigated its mass balance (part A) and absolute bioavailability (part B). In part A, six participants received tepotinib orally (498 mg spiked with 2.67 MBq [^14^C]-tepotinib). Blood, plasma, urine, and feces were collected up to day 25 or until excretion of radioactivity was <1% of the administered dose. In part B, six participants received 500 mg tepotinib orally as a film-coated tablet, followed by an intravenous [^14^C]-tepotinib tracer dose (53–54 kBq) 4 h later. Blood samples were collected until day 14. In part A, a median of 92.5% (range, 87.1–96.9%) of the [^14^C]-tepotinib dose was recovered in excreta. Radioactivity was mainly excreted via feces (median, 78.7%; range, 69.4–82.5%). Urinary excretion was a minor route of elimination (median, 14.4% [8.8–17.7%]). Parent compound was the main constituent in excreta (45% [feces] and 7% [urine] of the radioactive dose). M506 was the only major metabolite. In part B, absolute bioavailability was 72% (range, 62–81%) after oral administration of 500 mg tablets (the dose and formulation used in phase II trials). In conclusion, tepotinib and its metabolites are mainly excreted via feces; parent drug is the major eliminated constituent. Oral bioavailability of tepotinib is high, supporting the use of the current tablet formulation in clinical trials. Tepotinib was well tolerated in this study with healthy volunteers.

## Introduction

The mesenchymal–epithelial transition (MET) factor is a receptor tyrosine kinase with a single high-affinity ligand, hepatocyte growth factor (HGF) [1]. HGF binding leads to dimerization and autophosphorylation of MET, activating multiple downstream signaling pathways, including mitogen-activated protein kinase, phosphatidylinositol 3-kinase, signal transducer and activator of transcription protein, and nuclear factor kappa-light-chain-enhancer of activated B cells [1].

Oncogenic MET may not only initiate or promote tumorigenesis, but may also drive resistance to other therapies [2, 3]. Accordingly, exon 14 skipping and high-level amplification of *MET* are considered primary tumor-driving alterations in non-small cell lung cancer (NSCLC) [2, 4]. In addition, *MET* amplification is recognized as a secondary oncogenic driver alteration mediating resistance to epidermal growth factor receptor inhibition [2, 5]. Several inhibitors of the MET pathway are currently in development as potential antitumor therapies [2, 3, 6, 7].

Tepotinib (MSC2156119J) is an oral, highly selective, potent adenosine triphosphate-competitive inhibitor of MET, with >1000-fold selectivity for MET over 236 out of 241 other kinases tested, and >200-fold selectivity over the other five [8]. In the first-in-man trial, tepotinib doses of up to 1400 mg/day were administered in patients with solid tumors without reaching the maximum tolerated dose; 500 mg once daily was subsequently established as the recommended phase II dose (RP2D) based on a translational modeling approach [9]. Tepotinib alone or in combination has been shown to be active in various preclinical tumor models [8, 10–16]. It is currently being studied in clinical trials involving patients with hepatocellular carcinoma (NCT01988493, NCT02115373) [17, 18] and NSCLC (NCT01982955, NCT02864992, NCT03940703) [16, 19–22].

The purpose of the present study (EudraCT 2013-003226-86) was the characterization of the pharmacokinetics (PK), drug disposition, and metabolism of tepotinib in healthy volunteers. In part A of the study, [^14^C]-tepotinib was administered orally to evaluate the absorption, distribution, metabolism, and excretion of tepotinib. The availability of [^14^C]-labeled tepotinib, in conjunction with recent advances in the clinical use of microtracer doses and ultrasensitive bioanalytical methods [23], allowed the evaluation of the PK of tepotinib after intravenous (IV) dosing and its absolute bioavailability in part B of this study. For orally administered drugs such as tepotinib, characterization of the PK profile following IV dosing is of highest value for correct interpretation of distribution and elimination [24]. The combination of both investigations (mass balance and absolute bioavailability) in this study represented an opportunity to obtain a comprehensive understanding of the PK of tepotinib, supporting the ongoing formulation development and its phase II program.

## Materials and methods

### Study design

This was an open-label, single-center, phase I study in two parts, designed to investigate the mass balance, metabolism, and absolute bioavailability of tepotinib in two groups of six healthy male volunteers (EudraCT 2013-003226-86). Part A of the study was designed to evaluate the mass balance of tepotinib, identify major routes of elimination, determine the unbound fraction of tepotinib in plasma and its blood/plasma distribution, and identify and quantify metabolites of tepotinib in plasma, urine, and feces. Part B was designed to determine the absolute bioavailability of the phase II tablet formulation of tepotinib.

#### Part A

In part A of the study, six healthy volunteers received a single oral dose of 498 mg tepotinib spiked with 2.67 MBq [^14^C]-tepotinib (five capsules) on day 1. Participants were hospitalized from day 1 to day 16 of the study, and further assessed on days 19, 22, and 25 (trial end). Participants fasted for at least 10 h overnight prior to oral tepotinib administration, taken with a high-calorie, high-fat-content breakfast. Blood samples for quantitation of tepotinib and total radioactivity in blood and plasma were taken pre-dose and at 0.25, 0.5, 1, 1.5, 2, 3, 4, 6, 8, 10, 12, 16, 24, 30, 34, 48, 60, 72, 96, 120, 144, 168, 192, 216, 240, 264, 312, 360, 432, and 576 h after dosing. Separate blood samples were taken pre-dose and at 4, 12, 24, 60, 120, and 240 h post-dose for metabolite identification, and at 4, 8, 12, and 24 h post-dose for determination of the unbound fraction of tepotinib in plasma. Pre-dose feces and urine samples were collected on day 1 and day 2, respectively. Urine was collected at 4- to 12-h intervals on day 1 and every 24 h thereafter up to day 16. Feces were collected every 24 h up to day 16. Provision was made to collect further samples up to day 39 if >1% of the administered dose was detectable in the total (urine and feces) excreta per day on two consecutive measurements.

#### Part B

On day 1 of part B, six healthy volunteers received a single oral dose of 500 mg tepotinib (one tablet), followed by a delayed IV bolus injection of [^14^C]-tepotinib (53–54 kBq) 4 h later to cope with the risk of non-linear PK of the IV tracer dose [23]. Participants fasted for at least 10 h overnight prior to oral tepotinib administration, which was taken with a high-calorie, high-fat-content breakfast. Participants were hospitalized from day 1 to day 7 of the study, and further assessed on days 9, 11, and 14 (study end). Blood samples were taken pre-dose and at 0.25, 0.5, 1, 1.5, 2, 3, 4 (just before IV [^14^C]-tepotinib administration), 6, 8, 10, 12, 16, 24, 30, 34, 48, 60, 72, 96, 120, 144, 192, 240, and 312 h after oral tepotinib administration. Blood samples for the determination of [^14^C]-tepotinib were taken at 4 h (pre-IV dose) and 4.05, 4.25, 4.5, 5, 5.5, 6, 7, 8, 10, 12, 14, 16, 20, 28, 34, 38, 52, 64, 76, 100, 124, 144, 192, 240, and 312 h, in relation to the oral tepotinib administration.

### Participants

Eligible participants were healthy male volunteers aged 18–55 years with a body mass index (BMI) 18–30 kg/m^2^ and body weight >50 kg. Other inclusion criteria were: no tobacco smoking for ≥6 months before screening and body temperature, blood pressure, and pulse rate within normal ranges. Individuals were excluded from the study if they had any medical condition likely to interfere with study outcomes/assessments, history of clinically relevant disease, any clinically relevant abnormality in screening laboratory parameters/electrocardiogram (ECG), treatment with metabolic enzyme inhibitors/inducers, positivity for hepatitis B surface antigen, hepatitis C virus antibody or human immunodeficiency virus antibody, use of prescription-only medicine/over-the-counter medication within 2 weeks of study initiation, or excessive consumption of xanthine-containing drinks.

### Study medication and selection of doses

Oral tepotinib (MSC2156119J, Merck KGaA, Darmstadt, Germany) was formulated as 100 mg film-coated tablets. [^14^C]-tepotinib, radiolabeled on one carbon per molecule (specific activity, 54.6 mCi/mmol; radiochemical purity, 99.7%), was synthesized by Quotient Bioresearch (Rushden, UK). For part A, tablets were crushed and hard gelatin capsules were prepared to contain approximately 100 mg tepotinib and 0.5 MBq [^14^C]-tepotinib (~117 μg). For the absolute bioavailability part, a solution for injection was prepared to contain 0.05 MBq [^14^C]-tepotinib (~11.7 μg) per 1 mL of solution. The definition of the dose of oral [^14^C]-tepotinib required for determination of mass balance and quantification of tepotinib metabolites was based on preclinical data obtained following oral administration of [^14^C]-tepotinib to pigmented rats. For dosimetry calculations, the assumption was made that the levels of uptake and retention by respective tissues were the same in man as in experimental animals. Administration of 2.5 MBq (70.1 μCi, 0.585 mg) [^14^C]-tepotinib chosen for part A of this trial was expected to lead to a radiation burden of 0.5 mSv, which is below 1 mSv, as required by the International Commission on Radiological Protection (ICRP) Category IIa. The dosimetry calculation was carried out by the United Kingdom’s Health Protection Agency Centre for Radiation, Chemical and Environmental Hazards.

The single IV tracer dose of approximately 11.7 μg (in 1 mL) of [^14^C]-tepotinib (equivalent to 50 kBq [1.4 μCi]), used in part B of the trial to determine the absolute oral bioavailability, was in keeping with regulations regarding tracer doses set forth in ICH M3(R2) [25]. As this dose was expected to lead to a radiation burden below 0.1 mSv, which complies with the ICRP Category I limit of 0.1 mSv, no separate dosimetry was carried out for the IV tracer dose.

The RP2D of tepotinib of 500 mg was chosen to determine PK characteristics at a dose strength that most likely represents the therapeutic dose.

### Analytical methods

Bioanalysis of total plasma concentrations of unlabeled tepotinib was performed by Quintiles (Ithaca, New York, USA) using a validated liquid chromatography–tandem mass spectrometry (LC-MS/MS) method. In brief, the isotopically labeled internal standard [^2^H]-MSC2156119A was added to 20 μL K_2_-ethylenediaminetetraacetic acid (EDTA) plasma. Tepotinib was extracted by protein precipitation with acetonitrile/water (20 + 80, v/v). Samples were centrifuged and 10 μL aliquots of the supernatant were injected onto an Acquity Charged Surface Hybrid™ (CSH) C_18_ column (2.1 × 50 mm; 1.7 μM; Waters, Milford, Massachusetts, USA). Separation was carried out using a mobile phase gradient (eluent A: water/formic acid [100 + 1, v/v]; eluent B: acetonitrile/formic acid [100 + 1, v/v]) with a flow rate of 0.6 mL/min. Detection was performed in the positive ionization mode on an AB Sciex API-4000 triple quadrupole mass spectrometer (MS; AB Sciex, Framingham, Massachusetts, USA) with selected reaction monitoring (m/z, 493.3–112.1 for tepotinib and 496.3–115.1 for its deuterated internal standard). The lower limit of quantification (LLOQ) was 5.00 ng/mL, and the calibration standard responses were linear over the range between 5.00 and 2500 ng/mL using a weighted (1/x^2^) linear regression.

The determination of unbound tepotinib plasma concentrations, following equilibrium dialysis, was performed by Quintiles (Ithaca, New York, USA) using previously qualified LC-MS/MS analytical methods. Plasma (300 μL) was dialyzed against 100 mM sodium phosphate buffer (pH 7.4) in an orbital shaker at 37 °C for 18 h. The deuterated internal standard [^2^H]-MSC2156119A and 100 mM sodium phosphate buffer (pH 7.4) were added to 5 μL of the dialyzed samples and centrifuged. Supernatant (30 μL) was diluted with acetonitrile/water (50 + 50, *v*/v) and centrifuged, and 5–20 μL aliquots were injected onto an XBridge C_18_ column (2.1 × 20 mm; 5 μm; Waters, Milford, Massachusetts, USA). Separation was carried out using a mobile phase gradient (eluent A: 5 mM ammonium bicarbonate in water; eluent B: 5 mM ammonium bicarbonate in methanol) with a flow rate of 1.2 mL/min. Mass spectrometric detection of tepotinib and its internal standard was performed as described above. The LLOQ was 0.015 ng/mL in plasma dialysates and 1.00 ng/mL in plasma. Calibration standards between 0.015 and 30.0 ng/mL (plasma dialysates), and between 1.00 ng/mL and 1000 ng/mL (plasma) were calculated by weighted (1/x^2^) quadratic regression analysis, respectively.

Total radioactivity in blood, plasma, urine, and feces samples, and quick counts in urine and feces samples were determined at the bioanalytical laboratory of PRA (Assen, The Netherlands) using liquid scintillation counting (LSC). Scintillation cocktail Ultima Gold™ (5 mL; Perkin Elmar, Waltham, Massachusetts, USA) was directly spiked into 250 μL plasma and 1000 μL urine samples, respectively, whereas 300 μL aliquots of blood samples were solubilized and decolorized before adding 18 mL of the scintillation cocktail prior to LSC. Feces samples were (quantitatively) homogenized with one or two weight equivalents of water, and an accurately weighted aliquot of approximately 500 mg of the homogenate was dried at 50 °C for at least 3 h. After addition of 100 μL combustion aid, the dried samples were combusted in a sample oxidizer model 307 (Perkin Elmer, Waltham, Massachusetts, USA) using 7 mL CarboSorb^®^ E as an absorber agent for carbon dioxide. At the end of the combustion cycle, the absorber was mixed with 13 mL of the scintillant PermaFluor^®^ E. All scintillation counting using either low-level or normal count modes was carried out in duplicate using a Tri-Carb 3100 TR liquid scintillation analyzer (Perkin Elmer, Waltham, Massachusetts, USA), in compliance with analytical procedures validated by the PRA.

The accelerator mass spectrometry (AMS) analysis of [^14^C]-tepotinib in plasma samples in part B of the trial was performed by Eckert & Ziegler Vitalea Science (Davis, California, USA) using a validated method that involves isolation of the analyte by ultra-high-performance liquid chromatography (UPLC) fractionation, followed by the quantification of the ^14^C content in collected fractions using AMS [26]. Aliquots (300 μL) of K_3_-EDTA plasma samples were fortified with excess (nanograms) of unlabeled tepotinib, prior to extraction in order to create uniform total concentration levels of the analytes in all study samples, and to eliminate the potential for concentration-dependent recovery in the method. Following protein precipitation and further chemical steps, 50 μL of the reconstituted extracts were injected onto a Waters Acquity UPLC instrument equipped with a CSH fluoro-phenyl analytical column (3.0 × 100 mm; 1.7 μm; Waters, Milford, Massachusetts, USA) and an ethylene bridged hybrid C_18_ (2.1 × 5 mm; 1.7 μm; Waters, Milford, Massachusetts, USA) pre-column. Separation of [^14^C]-tepotinib from potentially interfering metabolites or degradants that may have been present in the study samples was carried out using a mobile phase gradient (eluent A: 5 mM ammonium acetate in water; eluent B: acetonitrile) with a flow rate of 0.75 mL/min. Liquid chromatography fractions corresponding to tepotinib were collected and prepared for AMS measurement, graphitized with a known amount of carrier carbon using a two-step process that involved oxidation to gaseous CO_2_ followed by reduction to solid, elemental graphite. Samples were then introduced into the BioMICADAS AMS instrument (PSI/ETH, Zurich, Switzerland). The LLOQ was 0.0883 pg/mL.

### Metabolite profiling and identification

Metabolite profiling and structure elucidation in plasma, urine, and feces were performed at the Institute of Drug Metabolism and Pharmacokinetics, Merck KGaA, Grafing, Germany, using a radiometric UPLC method coupled to a high-resolution MS. For urine and feces, metabolites were identified in a single pooled sample per matrix and subject (urine pool of 4–120 h and feces pool of 0–192 h), that covered at least 90% of the total radioactivity of the respective matrix excreted. Intervals with low radioactivity were not used for the generation of the pooled samples to avoid dilution, and to increase the signal-to-noise ratio of the respective chromatograms.

The metabolite structure elucidation was performed using the linear trap quadrupole Orbitrap XL hybrid Fourier transform (FT) MS (Thermo Scientific, Dreieich, Germany) coupled to a Waters Acquity UPLC system. Full-scan FT spectra were acquired for measurement of the masses of molecular ions of parent and metabolites. MS^2^ fragmentation spectra were acquired after high-energy collisional dissociation of the appropriate molecular ions. Allocation and confirmation of the proposed metabolites to the radioactive peaks were performed using an API4000 QTrap (AB Sciex, Darmstadt, Germany) MS with simultaneous splitting using a fraction collection system (CTC HTS PAL Fraction Collector, Axel Semrau, Sprockhövel, Germany), and offline radioactive measurement using a Topcount NXT^®^ microplate scintillation counter (PerkinElmer, Rodgau, Germany).

### Pharmacokinetic analyses

The PK parameters of tepotinib, total radioactivity, and metabolites were evaluated by standard non-compartmental methods using the software Phoenix WinNonlin (v.6.3, Certara INC, Princeton, New Jersey, USA). The maximum plasma concentration (C_max_) and time to reach maximum concentration (t_max_) were derived directly from the plasma concentration–time data. Lag time (t_lag_) was the time prior to the first measurable (non-zero) concentration. The area under the plasma concentration–time curve from 0 to t (last measurable concentration; AUC_0–t_) was calculated using a mixed log–linear trapezoidal method. Area under the plasma concentration–time curve until the last sampling time (AUC_all_), which corresponds to AUC_0–240 h_ within the observation period, was used for metabolite calculations. Amounts excreted in urine, feces, and total (urine + feces) were calculated as the sum of amounts (concentration multiplied by volume) for each collection from time 0 to time t. The terminal rate constant (λ_z_) was used to estimate the terminal half-life (t_½_) (ln 2/λ_z_), and the area under the concentration–time curve from time 0 to infinity (AUC_0–∞_). Total body clearance (CL) and apparent body clearance (CL/f) were determined by dose/AUC_0–∞_, and total volume of distribution (V_z)_ and apparent volume of distribution (V_z_/f) were calculated by dose/(AUC_0–∞_*λ_z_) after IV and oral administration, respectively. Volume of distribution of the central compartment (V_c_) was derived by dividing the dose by the initial drug plasma concentration after IV administration. Absolute bioavailability was calculated as F = (AUC_oral_/dose_oral_)/(AUC_IV_/dose_IV_) × 100. The ratios (%) AUC_tepotinib or metabolite_/AUC_total radioactivity_ were calculated as a percentage of the molar AUCs.

### Safety assessment

Treatment-emergent adverse events (TEAEs), changes in laboratory tests, ECG, vital signs, and physical examinations were assessed and graded using the National Cancer Institute Common Terminology Criteria for Adverse Events (NCI-CTCAE) version 4.0. All safety endpoints across both parts of the study were analyzed descriptively.

## Results

### Participants

A total of 12 male healthy volunteers were enrolled into the study, starting on December 11, 2013; the last participant’s final visit took place on May 2, 2014. In part A (*n* = 6), median age was 45.0 years (range, 25–54 years), and median BMI was 27.0 kg/m^2^ (range, 20.4–28.9 kg/m^2^). Five participants were Caucasian and one was of mixed parentage Caucasian/black or African/American. In part B (*n* = 6), median age was 22.5 years (range, 21–24 years), median BMI was 22.85 kg/m^2^ (range, 21.1–25.7 kg/m^2^), and all participants were Caucasian. All volunteers in both parts of the trial received treatment as scheduled.

#### Part A

### Mass balance and routes of excretion

**Fig. 1:**
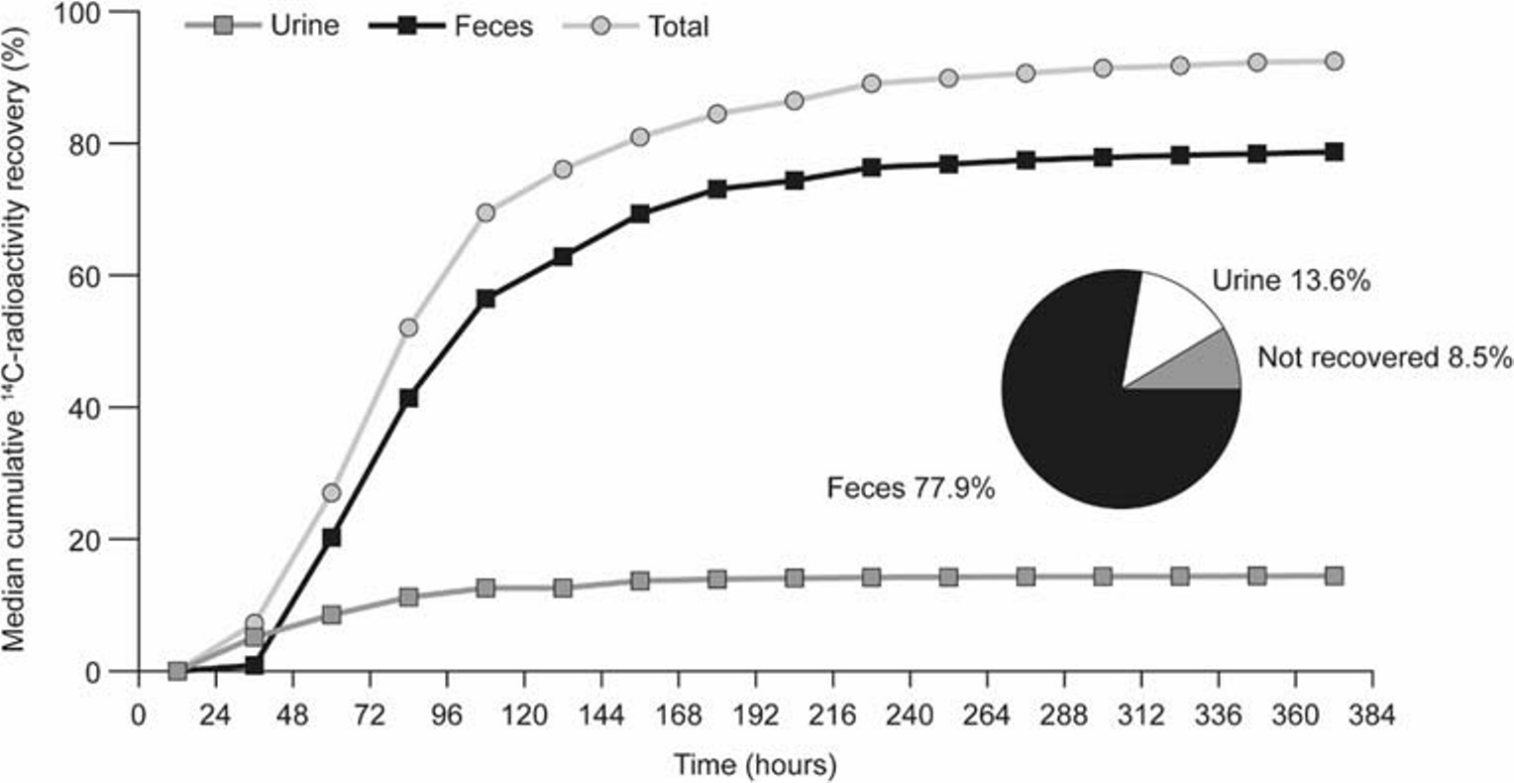
Median cumulative recovery of [^14^C]-radioactivity in urine and feces and combined (total). Pie chart shows respective geometric mean values

Median recovery of total radioactivity in excreta (feces plus urine) was 92.5% (range, 87.1–96.9%) of the orally administered [^14^C]-tepotinib dose (Fig. 1). Radioactivity was predominantly excreted in feces (median, 78.7%; range, 69.4–82.5%), and only to a minor extent in urine (median, 14.4%; range, 8.8–17.7%; Table 1). In feces, first radioactivity was detected after 24–48 h, and excretion was noted in all participants up to 360 h post-dose. In urine, radioactivity was observed within the first collection period of 4 h, and up to 360 h after dosing (with values below the LLOQ at 312 and 336 h after dosing in two participants). For all subjects, collection was stopped already at day 16 because <1% of the [^14^C]-tepotinib dose was recovered in excreta on two consecutive measurements, i.e. days 15 and 16.

**Table 1:** Individual amounts of relative radioactivity excreted shown as percentage of total radioactive dose of [^14^C]-tepotinib in urine, feces, and combined (total)

Subject	Ae_0–t urine_ (%)	Ae_0–t feces_ (%)	Ae_0–t total_ (%)
1	8.80	79.48	88.28
2	13.68	77.92	91.60
3	17.72	69.40	87.12
4	16.47	76.89	93.36
5	11.93	82.53	94.46
6	15.17	81.76	96.93
Min	8.80	69.40	87.12
Median	14.43	78.70	92.48
Max	17.72	82.53	96.93
Geometric mean	13.61	77.87	91.90

Ae_0–t_, amounts excreted in urine, feces, and total (urine + feces), calculated as the sum of amounts (concentration × volume) for each collection from time 0 to time t

### Oral PK of tepotinib and [^14^C]-tepotinib-related total radioactivity

**Fig. 2:**
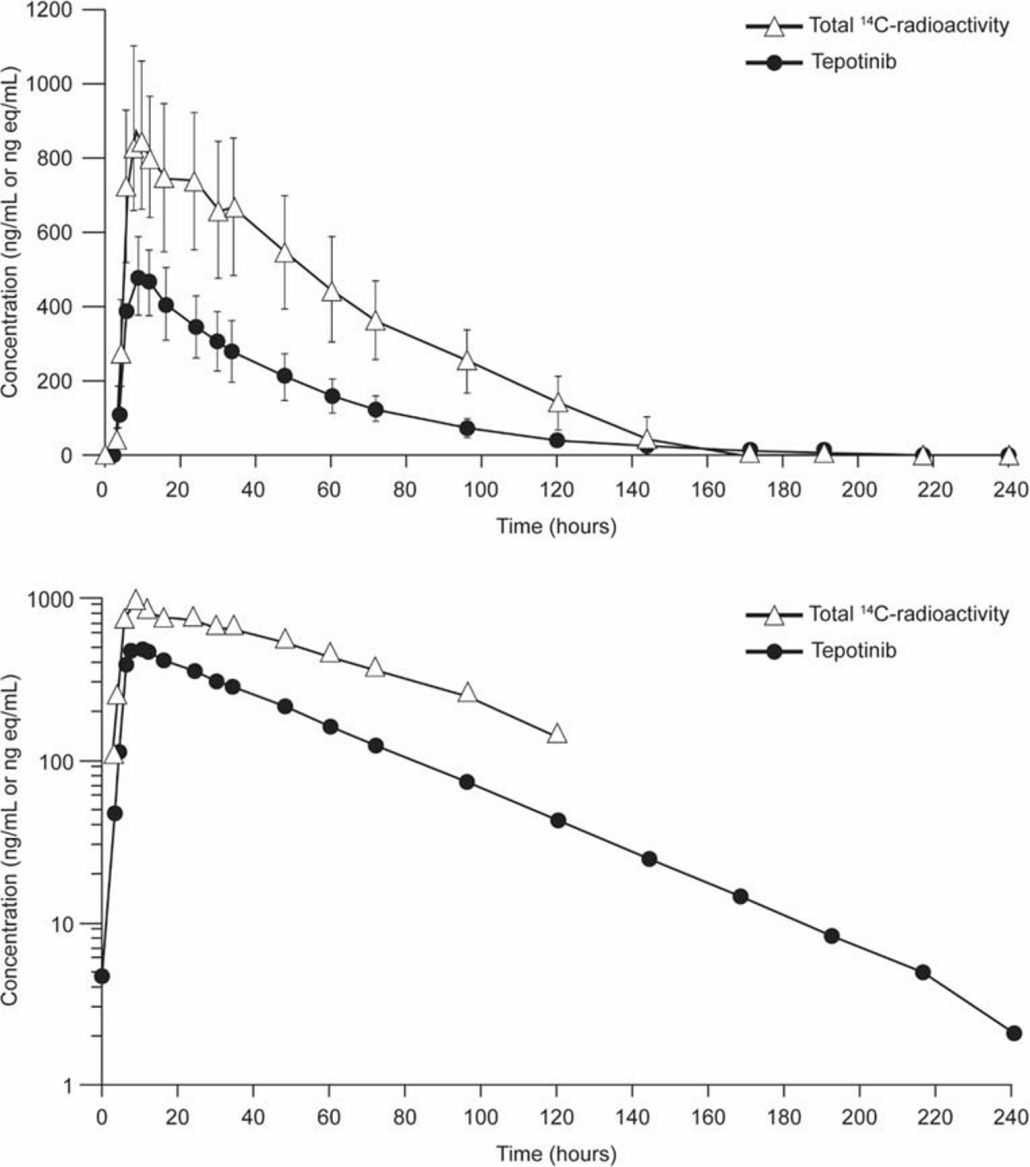
Mean plasma concentration–time curve for tepotinib and total radioactivity (linear scale [top panel] and log-linear scale [bottom panel]) (*n* = 6)

Concentrations of tepotinib and total radioactivity in plasma increased slowly, with a median t_max_ of 9 h, and a median t_lag_ of 2 and 2.5 h, respectively (Table 2). Comparison between the plasma concentration–time profile of total radioactivity, and tepotinib (as shown in Fig. 2) suggests the formation of circulating metabolites with elimination rate-limited kinetics – a conclusion that is supported by the longer apparent t_½_ of 38 h for total radioactivity versus 33 h for tepotinib. Accordingly, higher exposure values for AUC_0–t_, AUC_0–∞_, and C_max_, and smaller values for CL/f and V_z_/f, were observed for total radioactivity compared with tepotinib (Table 2). A median of only 14.4% of the radioactive dose was excreted via the kidneys. This is consistent with the small geometric mean value of 1.2 L/h estimated for the renal clearance of total radioactivity, which indicates that renal elimination contributes to the overall clearance of tepotinib, and its metabolites only to a minor extent. The blood:plasma ratio of total radioactivity was approximately 0.8 over time, and the respective concentration–time profile observed for total radioactivity in blood paralleled with that observed in plasma measured at the same time points. This blood:plasma ratio is also noted for C_max_ and AUC (Table 2), indicating that some radioactivity is distributed into blood cells when taking into account the median hematocrit of 0.445 observed in the six healthy volunteers on day 1 pre-dose. The unbound fraction of tepotinib in plasma was evaluated at four time points around t_max_, (i.e. 4, 8, 12, and 24 h after oral administration), with median values ranging between 1.8% and 2.4%. The lowest and highest free fractions observed in individual participants were 1.65% and 3.25%, respectively. The ex vivo protein binding data are in agreement with results derived from in vitro studies, in that the unbound fraction of tepotinib in human plasma ranged from 1.6–3.4% over the concentration range between 0.3 and 10 μM [27]. Tepotinib has been shown to bind primarily to plasma proteins [28]; human serum albumin and α-acid glycoprotein were previously identified as the main binding partners [29].

**Table 2:** PK parameters of tepotinib in plasma and total radioactivity in plasma and blood following administration of a single oral dose of 498 mg tepotinib spiked with 2.67 MBq [^14^C]-tepotinib in five capsules (geometric mean data; *n* = 6)

PK parameter^a^	Units	Tepotinib Plasma	Total radioactivity^b^ Plasma	Total radioactivity^b^ Whole blood
C_max_	ng/mL or ng eq/mL	487.0 (22.1), 354.0–652.0	875.3 (24.6), 598.0–1149.0	716.6 (18.0), 541.0–820.0
t_max_	Hour	9.0, 6.0–12.0	9.0, 7.9–24.0	8.0, 7.9–12.0
t_lag_	Hour	2.0, 1.5–3.0	2.5, 2.0–4.0	2.5, 2.0–4.0
AUC_0–t_	ng/mL × hour or ng eq/mL × hour	23,184 (27.6), 14,599–29,381	51,971 (36.2), 27,443–76,178	40,379 (28.2), 24,303–54,697
AUC_0–∞_	ng/mL × hour or ng eq/mL × hour	23,471 (27.2), 14,886–29,650	60,134 (31.0), 34,033–81,399	46,063 (25.2), 29,174–60,029
t_½_	Hour	33.0 (9.3), 28.6–36.6	37.8 (14.5), 30.9–47.8	37.6 (15.5), 30.4–46.8
CL/f	L/hour	19.1 (27.2), 15.1–30.1	7.46 (31.0), 5.5–13.2	9.7 (25.2), 7.5–15.4
V_z_/f	L	909.0 (24.6), 700.3–1244.1	406.6 (38.2), 245.8–724.0	528.4 (22.5), 416.8–774.2

^a^Values are geometric mean (geometric CV%), followed by min–max, except for t_max_ and t_lag_, for which median values, followed by min-max, are provided

^b^Total radioactivity is expressed in units of nanogram-equivalents per milliliter (C_max_) or nanogram-equivalents × hours per milliliter (AUC)

AUC, area under the concentration–time curve; AUC_0-∞_, area under the concentration–time curve from time 0 to infinity; AUC_0–t_, area under the curve from 0 to t (last measurable concentration) hours; CL/f, apparent body clearance; C_max_, maximum plasma concentration; CV, coefficient of variation; PK, pharmacokinetic; t_½_, elimination half-life; t_lag_, lag time prior to the first measurable (non-zero) concentration; t_max_, time to reach maximum concentration; V_z_/f, apparent volume of distribution

### Metabolite identification and profiling in plasma and excreta

**Fig. 3:**
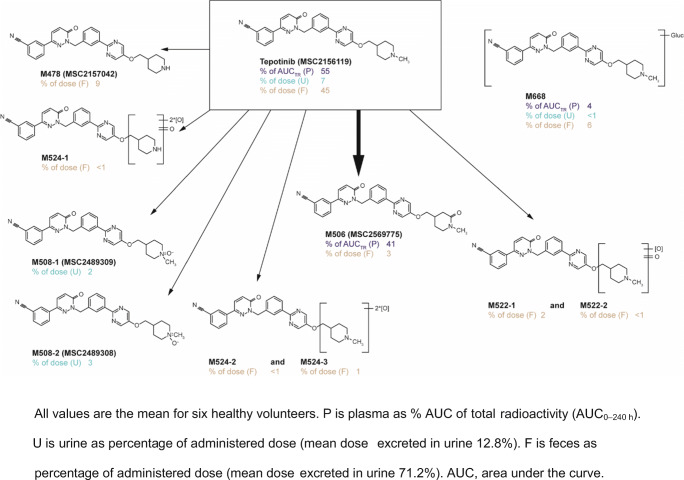
Proposed metabolic pathway for tepotinib. Structural formulae are postulates and have yet to be confirmed by nuclear magnetic resonance analysis and/or chemical synthesis, except for those proven by synthesis: racemic M506 (MSC2569775), M508–1 (MSC2489309), M508–2 (MSC2489308), and M478 (MSC2157042)

A total of ten phase I and phase II metabolites were detected. This included the chiral metabolite M506, which was elucidated to be formed by keto oxidation of the piperidine ring of tepotinib in the alpha position of the nitrogen. Of note, the formation of the respective enantiomers of this chiral metabolite was quantified in an additional analysis, applying an enantioselective LC-MS/MS method. M668 was likely formed by direct N-glucuronidation. Demethylation on the methyl-piperidine moiety formed metabolite M478. M524–1, −2, and − 3; M522–1 and − 2; and the two diastereomeric N-oxides M508–1 and − 2 were formed by further oxidation, or oxidation and demethylation. Figure 3 shows the proposed structures of tepotinib metabolites, including quantitative information in plasma, feces, and urine. In plasma, proposed structures could be allocated to all three radioactive peaks observed. These consisted of tepotinib and two metabolites, M506 and M668, which accounted for 55%, 41%, and 4% of the AUC_all_ of total ^14^C-radioactivity, respectively. M506 had a median t_max_ of 24 h (range, 24–60 h). Its apparent median t_½_ was 92 h (range, 66–145 h), based on data from four healthy volunteers. The minor plasma metabolite M668 had a median t_max_ of 12 h (range, 12–24 h) and an apparent t_½_ of 55.8 h, based on data from a single volunteer.

In feces, 93% of the recovered radioactivity could be structurally identified. The parent compound excreted in feces accounted for 45% of the total administered radioactive dose. The des-methyl metabolite M478 accounted for 9%, the direct N-glucuronide M668 for 6%, and the racemic metabolite M506 for about 3%. Altogether, the oxidized metabolites M524–1, −2, and − 3, and M522–1 and − 2 each accounted for ≤2% of the administered dose.

In urine, 91% of the recovered radioactivity could be allocated to chemical structures. Most prominent was the parent drug tepotinib, comprising 7% of the total radioactive dose. The N-oxide metabolites M508–1 and − 2 accounted for about 2% and 3% of the total administered dose, respectively.

Overall, 52% of the total radioactive administered dose excreted in feces or urine was associated with the parent compound and approximately 23% and 7% with phase I and phase II metabolites, respectively (Fig. 3).

#### Part B

### Oral and IV PK of tepotinib

**Fig. 4:**
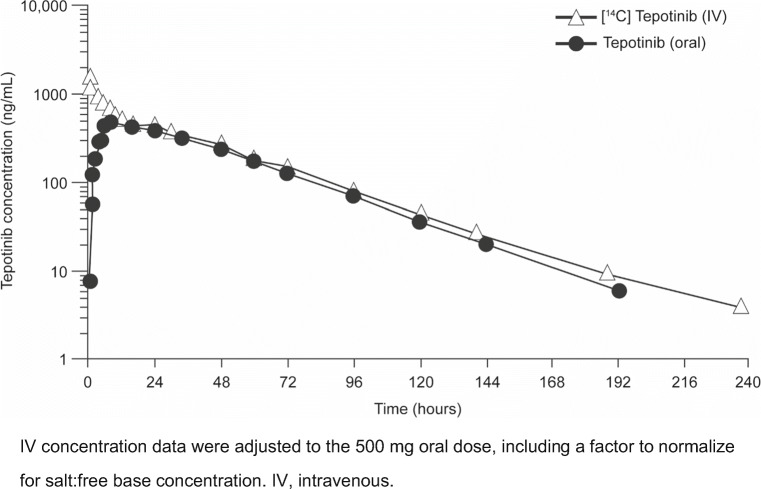
Mean plasma concentration–time curves for tepotinib after oral and IV administration (log-linear scale)

Six healthy volunteers received a single oral dose of 500 mg tepotinib followed by an IV bolus injection of [^14^C]-tepotinib (actual individual doses ranged from 53 to 54 kBq) 4 h later. Figure 4 suggests a triphasic decline of plasma concentrations, including a sharp drop during the first 15 min, followed by a second decline phase of up to 6–12 h before a final mono-exponential decrease in concentrations. The initial sharp decline in concentrations is mirrored by the small geometric mean value of 34.6 L estimated for V_c_, which indicates that tepotinib rapidly distributes out of the central compartment (Table 3). In comparison to V_c_, a much higher geometric mean value for V_z_ (573.6 L) was observed, which points to pronounced distribution of tepotinib into peripheral compartments. The geometric mean CL was 12.8 L/h, and a geometric mean elimination t_½_ of 31.2 h could be estimated from the terminal slope of the plasma concentration–time profile after bolus injection of the IV [^14^C]-tracer dose. After parallel single oral administration of 500 mg tepotinib in the current phase II tablet formulation, t_lag_ was 0.5 h and t_max_ was 10 h. Tepotinib concentrations declined in a mono-exponential fashion with an apparent t_½_ of 28.3 h, similar in range to the t_½_ values estimated after IV dosing. Geometric mean values for V_z_/f and CL/f of 726.0 L and 17.8 L/h, respectively (Table 3), were slightly larger after oral than after IV dosing.

**Table 3:** PK parameters in plasma following administration of a single oral dose of tepotinib (500 mg) and an IV tracer dose of [^14^C]-tepotinib (11.7 μg/mL, equivalent to 53–54 kBq [1.4 μCi])

PK parameter^a^	Units	[^14^C]-tepotinib (IV tracer)	Tepotinib (oral)
C_max_	ng/mL	8580.3 (36.4),^b^ 5816.0–15,946.5	555.7 (13.6), 469.0–675.0
t_max_	Hours	–	10.0 (3.0–10.0)
t_lag_	Hours	–	0.52 (0.25–2.0)
AUC_0–t_	ng/mL × hours	35,137.8 (7.8),^b^ 33,203.1–40,996.4	25,000.9 (16.9), 20,319–32,217
AUC_0–∞_	ng/mL × hours	35,316.9 (7.8),^b^ 33,370.6–41,206.0	25,300.2 (16.7), 20,615–32,559
t_½_	Hours	31.2 (13.3), 24.2–35.2	28.3 (16.5), 20.5–32.3
CL or CL/f	L/hours	12.8 (7.8), 10.9–13.5	17.8 (16.7), 13.8–21.8
V_c_	L	34.6 (44.3), 16.4–54.5	–
V_z_ or V_z_/f	L	573.6 (14.4), 463.7–659.0	726.0 (24.8), 550.0–969.4

^a^Values are geometric mean (geometric CV%), followed by min–max, except for t_max_ and t_lag_, for which median values, followed by min–max, are provided

^b^To allow comparisons in this table, exposure parameters C_max_, AUC_0–t_, and AUC_0–∞_ estimated after IV tracer administration were individually adjusted to the 500 mg oral dose, including a factor to normalize for salt:free base concentration

AUC_0–∞_, area under the concentration–time curve from time 0 to infinity; AUC_0–t_, area under the curve from 0 to t (last measurable concentration) hours; CL, total body clearance; CL/f, apparent body clearance; C_max_, maximum plasma concentration; CV, coefficient of variation; IV, intravenous; PK, pharmacokinetic; t_½,_ elimination half-life; t_lag_, lag time prior to the first measurable (non-zero) concentration; t_max_, time to reach maximum concentration; V_c_, volume of distribution of the central compartment; V_z_, total volume of distribution; V_z_/f, apparent volume of distribution

### Bioavailability

At the current recommended phase I dose of tepotinib of 500 mg/day [9, 30], and using the tablet formulation being administered in ongoing clinical phase II trials, tepotinib had a geometric mean absolute bioavailability of 72% (range, 62–81%), with low inter-individual variability (geometric CV, 10.8%).

### Safety

The single oral dose of 500 mg tepotinib was well tolerated. Nine of 12 healthy volunteers experienced 19 TEAEs, 17 of which were grade one in severity (according to NCI-CTCAE). The grade two TEAEs were abdominal pain and presyncope. All TEAEs had resolved by the end of the study, except for one incidence of grade one arthralgia reported by a subject in part B; however, this was considered to be unrelated to the study medication. Eight TEAEs were judged to be treatment related; diarrhea and abdominal pain were reported in two patients each. There were no withdrawals due to TEAEs and neither deaths, serious adverse events, nor other significant adverse events were reported.

## Discussion

This phase I, open-label study was conducted to investigate the mass balance, to identify and profile metabolites, and to assess the absolute bioavailability of tepotinib in two groups of healthy male volunteers. During early stages of clinical development, knowledge of absolute bioavailability is often missing, although this information can be extremely helpful to guide development of oral drugs [30]. The availability of [^14^C]-labeled material, as part of a standard mass balance study, is an opportunity to investigate absolute bioavailability in parallel with minimal additional effort [23]. The use of a tracer IV dose reduces formulation work and, in line with ICH M3(R2) [31], may also not require generation of additional preclinical IV data to cover administration in humans. To optimize the approach, both investigations can even be performed in the same group of participants [32]. However, this approach was not applied in this study, due to practical considerations related to uncertainties regarding the duration of the excreta collection periods in part A of the study, which turned out to be shorter than initially expected.

Following single oral administration of 498 mg tepotinib spiked with 2.67 MBq [^14^C]-tepotinib to male healthy volunteers, the overall median recovery of the radioactive dose in excreta was 92.5%. For a study to provide appropriate information about the excretion of a compound, the European Medicines Agency recommends that recovery of radioactivity in urine and feces should be at least 90% of the radioactive dose [33]. Therefore, we conclude that the study provides a good and reliable assessment of the mass balance of tepotinib and, consequently, of its elimination pathways.

Radioactivity was predominantly recovered in feces, accounting for 78% of the radioactive dose, while only approximately 14% of radioactivity was excreted via urine. Unchanged tepotinib was identified as the main source of radioactivity, accounting in total for 52%, and in feces for 45%, of the radioactive dose. Recovery of a high fraction of unchanged drug in feces, after oral administration, in a mass balance study often suggests incomplete absorption from the gastrointestinal tract. However, the advantage of the design of this study is that the results of part B provided insight into gastrointestinal drug absorption. In this case, the high absolute bioavailability of tepotinib of 72%, determined in part B, pointed to a high absorbed fraction of orally dosed tepotinib, which indicates that incomplete absorption was not the main root cause for the high level of radioactivity associated with unchanged tepotinib recovered in feces. In support of this, preclinical results from an excretion study with [^14^C]-tepotinib in bile-canaliculated rats indeed showed that a predominant fraction of radioactivity is excreted unchanged via the bile. Since tepotinib is known to be a substrate of the drug efflux transporter P-glycoprotein [29], active transport of the parent drug (via bile) may also represent a relevant elimination pathway of tepotinib in humans. Interestingly, none of the individual concentration–time profiles derived from tepotinib or total radioactivity showed any secondary peaks. This may suggest that neither tepotinib nor its metabolites (potentially released during its intestinal passage, e.g. formed by hydrolysis of the [direct N-]glucuronide M668 or reduction of the N-oxide M508), are re-absorbed at relevant quantities. However, we cannot exclude the absence of distinct secondary peaks being due to a slow absorption rate.

Ten metabolites of tepotinib were identified in this clinical trial. The identification of metabolites in plasma and excreta was almost complete, and far above the recommended threshold of 80% [25]. Overall, the influence of metabolism on the elimination of tepotinib appears to be modest. Only approximately 30% of the radioactive dose can be related to the structurally identified metabolites in excreta; a value much smaller than the 52% related to unchanged drug. Although the drug-metabolizing enzymes involved in the formation of all of the identified metabolites have not yet been completely determined, it can nevertheless be concluded from the data that the contribution of a single enzyme, e.g. cytochrome P450 isoforms or uridine diphosphate-glucuronosyltransferases, to the sum of either phase I or II metabolic pathways does not account for more than 25% of tepotinib’s overall elimination. In addition, the low total clearance observed after IV administration in relation to human liver blood flow further suggests that tepotinib is subject to only minor pre-systemic metabolism (hepatic and/or intestinal) following oral administration. Based on the results of this study, we consider that there is a low risk for clinically significant alterations to the PK of tepotinib, either by inhibition or induction of drug-metabolizing enzymes through the use of concomitant medications (drug–drug interactions) or by functional polymorphisms of these enzymes.

M506 was the only major metabolite detected in plasma (i.e. >10% of total drug-related exposure, as specified in current guidelines) [25, 31, 34]. While M506 accounted for 41% of the total radioactivity exposure in plasma, it only accounted for 3% of radiolabeled drug in excreta, which, also in the absence of any related secondary metabolite, indicates that this is not an important route of elimination (Fig. 3). Interestingly, M506 was not identified in previous in vitro metabolism studies with [^14^C]-labeled tepotinib using hepatocytes from multiple species, including humans. The trace amount of M506 and its slow formation rate in hepatocytes likely impeded its detection by LSC. The apparent discrepancy between the prominent appearance of this metabolite in the systemic circulation, and the small fraction excreted may be related to a number of factors. M506 was found to be highly protein-bound, with a low affinity for blood cells. Thus, a low volume of distribution, combined with a low clearance, might explain the relative over-representation of M506 in plasma. Further results from ongoing investigations will provide more information about the PK of this chiral metabolite, and will also contribute to a better understanding of the relevance of enantiomers of M506.

In summary, tepotinib has high absolute bioavailability (72%) with low inter-individual variability, providing consistent and predictable dose delivery, and relative insensitivity to factors that can increase variability, such as drug–drug interactions. This contrasts with many small-molecule kinase inhibitors that have poor oral bioavailability and, therefore, also have variable and less predictable PK [30]. Moreover, the late t_max_, in conjunction with the long t_½_ of tepotinib of 30 h, results in small peak–trough fluctuations at steady-state, and should enable continuous and constant inhibition of the MET receptor with once-daily dosing. Tepotinib has a large volume of distribution, indicating preferential partitioning into tissues, and indeed has been consistently found at higher concentrations in xenograft tumors than in the plasma of mice [8], which further supports its target inhibition. Overall, the PK of tepotinib as assessed in this study showed very favorable characteristics for further development in clinical trials.

In this study, a single 500 mg dose of tepotinib was well tolerated by healthy volunteers; although 75% experienced TEAEs, most were mild and resolved rapidly. Tepotinib has also been found to be well tolerated in phase I/Ib and phase II clinical trials in patients with solid tumors, which have further characterized its side-effect profile [9, 17, 18, 21, 35, 36]. Ongoing phase II trials in NSCLC and hepatocellular carcinoma will add further insight into the side-effect profile and efficacy of tepotinib [16–18, 20–22, 35].

## Conclusions

This study has demonstrated that the tablet formulation of tepotinib at the RP2D of 500 mg once daily has high oral bioavailability, supporting its use in phase II studies. Tepotinib exhibits low-to-moderate clearance and a high volume of distribution, resulting in a long plasma t_½_. Thus, its PK profile is considered favorable for once-daily dosing to ensure continuous target inhibition. Hepatic elimination is the major pathway in humans and tepotinib is primarily excreted unchanged via feces, and only to a minor extent via urine. Ten phase I and II metabolites were identified in total (plasma and excreta), with chiral M506 being the only major circulating metabolite that warrants further characterization. The results also indicate that the potential for drug–drug interactions affecting the PK of tepotinib is low, irrespective of whether concomitant medications induce or inhibit drug-metabolizing enzymes.
